# A cross-sectional pilot study assessing needs and attitudes to implementation of Information and Communication Technology for rational use of medicines among healthcare staff in rural Tanzania

**DOI:** 10.1186/1472-6947-14-78

**Published:** 2014-08-27

**Authors:** Jessica Nilseng, Lars L Gustafsson, Amos Nungu, Pia Bastholm-Rahmner, Dennis Mazali, Björn Pehrson, Jaran Eriksen

**Affiliations:** 1 Division of Clinical Pharmacology, Department of Laboratory Medicine, Karolinska Institutet at Karolinska University Hospital Huddinge, 141 86 Stockholm, Sweden; 2 Department of Computer Studies, Dar es Salaam Institute of Technology, Dar es Salaam, Tanzania; 3 School of Information and Communications Technology, KTH Royal Institute of Technology, Stockholm, Sweden; 4 Department of Health Care Development, Public Healthcare Administration, Stockholm County Council, Stockholm, Sweden; 5 Dept of hygiene and environmental medicine, Muhimbili University of Health and Allied Sciences, Dar es Salaam, Tanzania

**Keywords:** Access to medicines, Africa, Android tablet, Decision support, Health systems, ICT, iPad, Low-income countries, Rational use of medicines

## Abstract

**Background:**

In resource-poor countries access to essential medicines, suboptimal prescribing and use of medicines are major problems. Health workers lack updated medical information and treatment support. Information and Communication Technology (ICT) could help tackle this. The impact of ICT on health systems in resource-poor countries is likely to be significant and transform the practice of medicine just as in high-income countries. However, research for finding the best way of doing this is needed. We aimed to assess current approaches to and use of ICT among health workers in two rural districts of Tanzania in relation to the current drug distribution practices, drug stock and continuing medical information (CME), as well as assessing the feasibility of using ICT to improve ordering and use of medicines.

**Methods:**

This pilot study was conducted in 2010–2011, mapping the drug distribution chain in Tanzania, including problems and barriers. The study was conducted in Bunda and Serengeti districts, both part of the ICT4RD (ICT for rural development) project. Health workers involved in drug procurement and use at 13 health facilities were interviewed on use and knowledge of ICT, and their attitudes to its use in their daily work. They were also shown and interviewed about their thoughts on an android tablet application prototype for drug stock inventory and drug ordering, based on the Tanzanian Medical Stores Department (MSD) current paper forms.

**Results:**

The main challenge was a stable supply of essential medicines. Drug supplies were often delayed and incomplete, resulting in stock-outs. All 20 interviewed health workers used mobile phones, 8 of them Smartphones with Internet connection. The Health workers were very positive to the tablet application and saw its potential in reducing drug stock-outs. They also expressed a great need and wish for CME by distance.

**Conclusion:**

The tablet application was easily used and appreciated by health workers, and thus has the potential to save time and effort, reduce transportation costs and minimise drug stock-outs. Furthermore, the android tablet could be used to reach out with CME programs to health care workers at remote health facilities, as well as those in towns.

## Background

In resource-strained countries, particularly in sub-Saharan Africa (SSA), weak health systems are challenged by a disease burden that is high compared to the rest of the world [1]. Although up to 65% of health care costs in resource-poor countries are spent on medicines, compared to 7-30% in rich countries [2], poor access to life-saving essential medicines, counterfeit drugs as well as suboptimal prescribing and use of medicines are major problems [3,4]. Information and Communication Technology (ICT) could help tackle such problems, but to apply existing ICT and decision support systems from high-income countries in resource-strained countries may not achieve the required results [5]. Compared to richer countries most health facilities in SSA countries have little or no access to modern technical tools. However, several studies indicate that the impact of ICT on health systems in resource-strained countries will be significant and will transform the practice of medicine just as it has done in high-income countries [1,6,7].

There are several challenges to implementing ICT services for improving the quality of practices in health care institutions in resource-poor countries. A number of small technical pilot studies in healthcare institutions have been reported to be successful in resource strained countries, but existing over-burdened and sometimes corrupt public health care systems have made large scale implementations difficult [8]. According to The World Bank less than 14% of the population in Tanzania had access to electricity in 2009 [9], making it critical that ICT installations are based on renewable local techniques for energy supply. In 2004, the number of personal computers per 1,000 persons in Tanzania was 7,4 compared to 763 in Sweden [10]. However, mobile phone penetration passed 40% in SSA already in 2010 and continues to increase rapidly [11], offering other possibilities for introduction of ICT tools in both rural and urban settings.

ICT projects using mobile phone solutions have been successfully piloted in African countries for patient contacts with health care providers, distance consultations, disease surveillance, diagnostics, research networks and to provide better access to essential drugs and more [5,12-14]. An example of a successfully implemented ICT tool is the *“SMS for life”* project where mobile phone reports were used to redistribute antimalarial drugs from health facilities with a good drug stock to health facilities with drug shortage. During a pilot study in rural Tanzania the proportion of health facilities without stock of antimalarial drugs decreased from 78% to 26% [15]. Today *“SMS for life”* has been rolled out across Tanzania [16], with plans to expand the system to other drugs [17]. However, the impact of this type of information system to ensure good access to medicines has not been evaluated from a long-term perspective.

In Tanzania, the Medical Stores Department (MSD) distributes all drugs to public health facilities through nine zonal offices. MSD is still working towards long-term goals for improving sustainability of drug procurement, storage and distribution [18]. Integration of these three could potentially decrease distribution costs in Tanzania. Distribution within the system is carried out using an Integrated Logistic System (ILS) [19] through which every public health facility receives a yearly allowance for drugs and medical items. This allowance is not related to demographic factors of the catchment area population of the health facility, which results in uneven drug distribution due to vastly different population sizes and disease burden. The ILS is an improvement to the former system, where the government distributed essential drugs for the health facilities in pre-packed kits without considering individual health facility supply needs [20].

This study aimed to assess current approaches to and use of ICT among health care workers in two rural districts of Tanzania in relation to the current drug distribution practices, stock of essential medicines and continuing medical education (CME), as well as to assess the feasibility of using modern but simple ICT-technology integrated into the health-care system to improve the quality of drug chain management and CME in rural parts of Africa.

## Methods

This study adheres to the RATS guidelines (http://www.biomedcentral.com/authors/rats).

### Study area

This project was conducted in the Bunda and Serengeti districts, located in northern Tanzania and two of the poorest districts of the country. The districts were selected because of an ongoing collaboration between the district authorities and the study partners. Since 2003 the technical project Information and Communication Technology for Rural Development (ICT4RD) has been run by Tanzania Commission of Science and Technology (COSTECH), Dar-es-Salaam Institute of Technology (DIT) and the Royal Institute of Technology (KTH) [21-23]. The purpose is to design and validate a scalable method for establishment of broadband services in under-served areas. In 2006 a broadband network was successfully deployed connecting Bunda Township in Bunda district and Mugumu Township in Serengeti district in a network using an optical fibre backbone and wireless end-user connections, now connected to the new Tanzanian network backbone.

Bunda district has a population of about 375,000 [24] served by one district hospital, four health centres and 35 public dispensaries. Serengeti district, with a population of about 250,000 [24] has a designated district hospital, two health centres and 35 dispensaries. In Bunda and Serengeti districts, ordering of drugs and medical items is paper based, whereby forms are completed by each health facility quarterly and then brought to Mwanza MSD through the District Medical Officer (DMO). After a couple of months the goods are delivered to the district headquarters which then distributes them to the individual health facilities [25].

### Data collection

**Figure 1:**
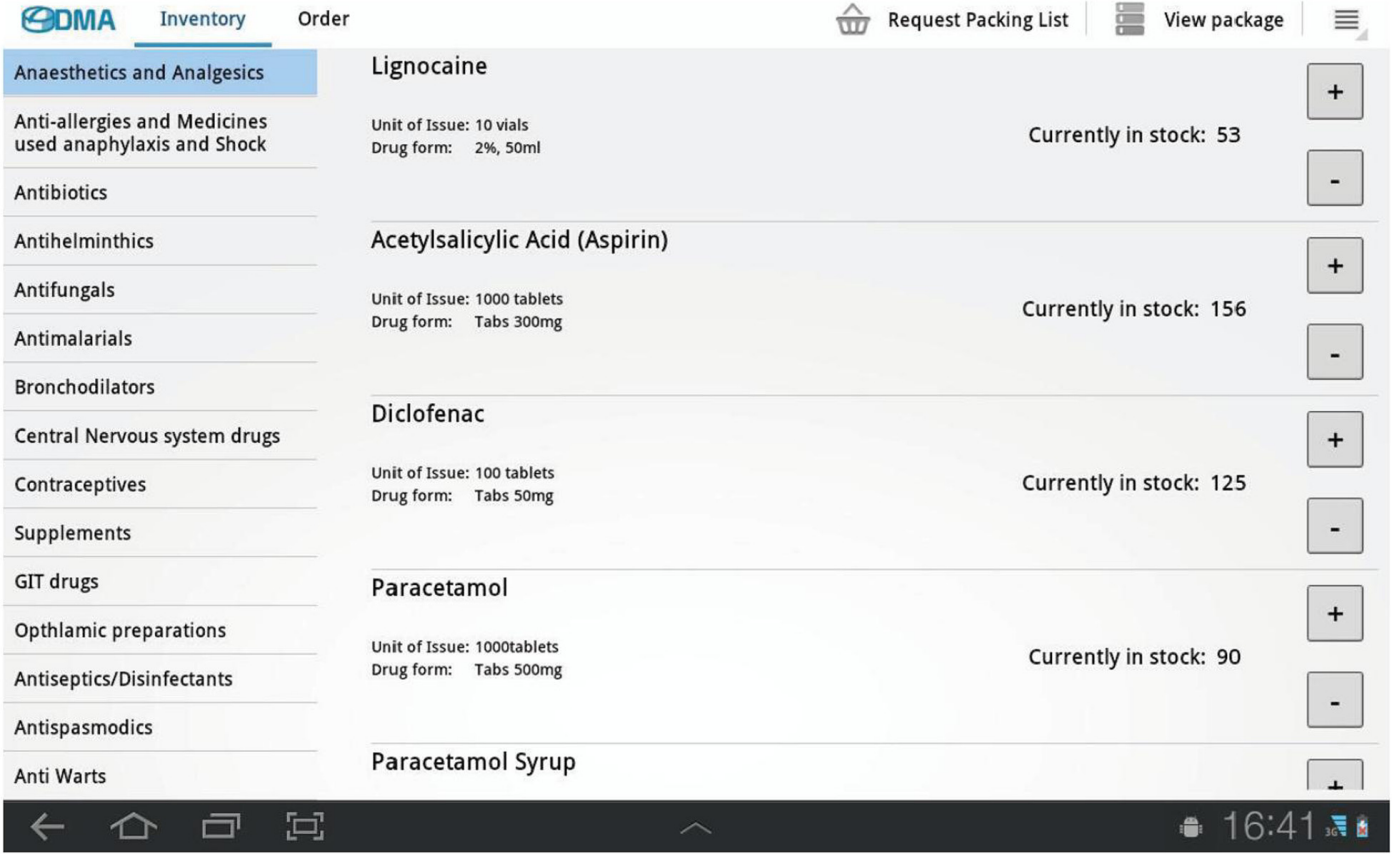
Screenshot of drug management application.

The data collection consisted of three parts:

1. Semi structured partly qualitative explorative interviews with 20 health care workers at 13 different health facilities at different levels of the health care system in Bunda and Serengeti districts to assess feasibility and usefulness of an application for securing drug distribution. Based on previous experience of phenomenographic studies, a sample of 20 respondents was considered large enough to capture the variability of the studied phenomenon [26,27]. Key informants within the healthcare and drug distribution system at the two designated district hospitals and at two associated health centres were chosen as the first 8 respondents using judgmental sampling [28]. The additional 12 respondents were identified using a snowballing approach [29]. The questionnaire (Additional file 1) included information about use of, approaches to, and interest in ICT, access to Continuing Medical Education (CME), information about drug supply for the facilities and the interviewees’ view of the drug distribution chain in Tanzania today, suggestions of improvement and how they thought ICT could be helpful. It was developed by the study team through several revisions and based on previous experiences from the field. The interviews were conducted at the informants’ workplace during two weeks in December 2011. Interviews took 20 to 60 minutes and were audio tape recorded and transcribed. All interviews were conducted in English by the first author (JN), but with a local interpreter to assist if language difficulties occurred.

2. Among the 20 respondents, 13 were the persons in charge at each of the included health facilities. During the interviews with these 13, additional questions were asked regarding the availability and use of electricity, computers, Internet connection and other relevant components of the infrastructure at the hospital, health centres and dispensaries in Bunda and Serengeti districts. Observations were also conducted regarding ICT infrastructure at the different health facilities.

3. A Drug Management Application, developed by KTH students [30], was demonstrated by JN to the ten interviewees in Bunda district, after the interviews (Figure 1). The application shown was a prototype of the now improved drug management application; it was installed on an Android device, a Samsung Galaxy tablet with both WiFi and mobile network interfaces. The application has two parts, one inventory and one ordering part. The Inventory part is used for viewing drug stock after incoming or consumed supply. The ordering part is used to add a new order or check a previous one. The drugs available in the list were the same as the paper based order form used at the health facilities. The layout of the application was simple; this prototype only had an English version. The interviewees were asked to try the application and then asked of their opinion of it, including its potential.

### Data analysis

In the transcription process of the qualitative part, redundant words were left out in the first written version. The transcribed text was read repeatedly by the researchers independently to gain an overall impression. The content was divided into units of analysis, referring to one specific interviewee, giving them a number and thereafter sorted under specific themes with correlated content. The themes were based on the questions and topics that arose from it. Thereafter the text was condensed so that the core of the content was preserved [31]. The themes were discussed and when opinion differed, whether about meaning or origin, we returned to the transcripts and sought evidence to establish consensus. This iterative process was used throughout the whole analysis, i.e. moving from the condensed description and back again. Quotations were selected to illustrate the informants’ views [31]. The analysis was carried out by a medical student (JN), a medical doctor (JE) and a behavioural scientist (PBR), all with previous experience of qualitative analysis.

The quantitative data were entered in an Excel sheet and analysed and proportions were calculated.

### Ethical considerations

A research clearance was granted from the Dar es Salaam Institute of Technology (DIT) (ref. No. BA/793/125/01/10), where it was deemed no further ethical review was necessary for this pilot study. This was then accepted locally both in Bunda and Serengeti districts, by the district executive director at each district headquarters. All interviewees were informed that their participation was voluntary and that they could withdraw from the study at any time, without any implications. This project was part of an undergraduate student project for medical students (JN) at Karolinska Institutet.

## Results

### Quantitative data

A total of 20 persons were interviewed at thirteen different health facilities. The health facilities were the two hospitals in Bunda and Mugumu, four health centres and seven dispensaries. Sociodemographics are presented in Table 1.

**Table 1:** Sociodemographics of the interviewees (n = 20) including age, gender, employment, workplace

**Characteristic**	**Description**	**N (total = 20)**
Location of participants	Serengeti district	10
	Bunda district	10
Age in years (span)	Median (range)	33,5 (23–57)
Gender	Male	16 (80%)
	Female	4 (20%)
Employment/Education	Nurse	3
	Medial assistant	2
	Clinical officer	6
	Assistant medical officer, Medical officer, Clinical dentist	5
	Pharmacist	4
Type of workplace	Hospital	9
	Health centre	4
	Dispensary	7
Time on current position	<1 year	5
	1-5 years	12
	>5 years	3

*Abbreviations*: *MA* Medical assistant, *CO* Clinical Officer, *AMO* Advanced Medical Officer, *MO* Medical Officer.

### Reported access to essential medicines

**Figure 2:**
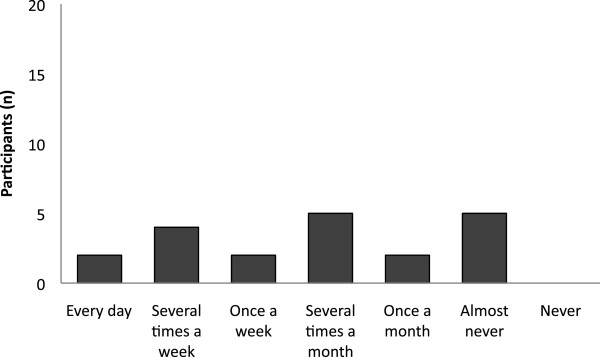
**Stock-outs of medicines.** This graph presents how often the participants experienced stock-outs of at least one 1^st^ line treatment at their health facility (n = 20).

**Figure 3:**
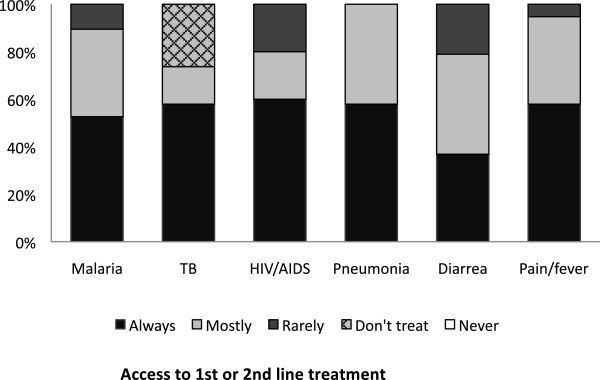
**Availability of essential medicines.** This figure shows how often the informants stated to have access to essential 1^st^ or 2^nd^ line treatment for specified diseases/conditions (100% = 20 participants).

The participants reported to have access to 1^st^ or 2^nd^ line treatment needed to treat their patients on average in 83% of the cases (50-100%). None said that they were never out of stock, and 10% (n = 2) reported that they were always out of stock of at least one 1^st^ line treatment (Figure 2). Figure 3 shows how often the interviewees estimated they had access to drugs for specified conditions at their health facility.

### Reported access to CME and to medical information

Workshops and conferences were reported as the most common way of continuing medical education (CME). These were attended one to four times per year and usually addressed one specific topic, e.g. sexual transmitted infections or HIV/AIDS. Ninety-four percent (n = 19) of interviewees also reported using medical literature or books for CME. These were mostly private books, but booklets/clinical practice guidelines from the Ministry of Health and Social Welfare (MoHSW) were also found at eight health facilities. Medical newsletters and journals from MoHSW, UNICEF, and WHO were received at workshops or through the district medical officer (DMO) a couple of times per year. As many as 56% (n = 11) of the interviewees also reported using the Internet for CME. All interviewees said they were interested or very interested in more CME and consultation through videoconferences.

### Reported ICT use and skills

**Figure 4:**
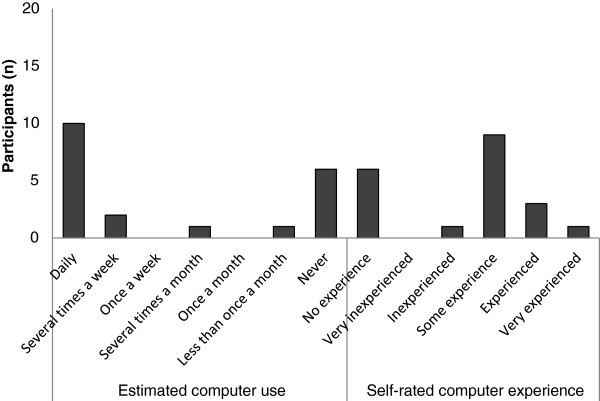
**Computer use and skill.** Figure describing how often the participant used a computer (left) and how they described their computer proficiency (n = 20).

**Figure 5:**
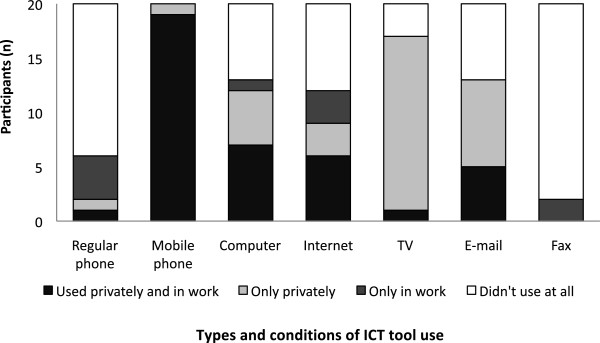
Use of different ICT tools among health care workers in Bunda and Serengeti districts (n = 20).

**Figure 6:**
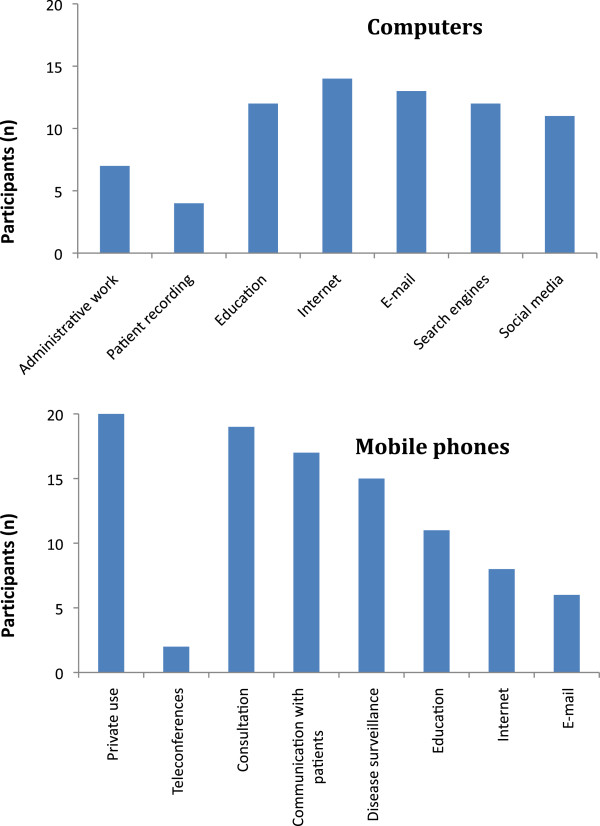
**Purpose of use of computers and mobile phones.** Purpose of use of computers and mobile phones as stated by informants (more than one option possible) (n = 20).

Mobile phones were the most commonly used ICT tool, used by all interviewees. Figure 4 shows how often participants used a computer and how they estimated their computer skills. Figure 5 shows what types of ICT tools interviewees used and whether they used them for work or privately. All interviewees used mobile phones and 12 used the Internet. Eight interviewees (40%) reported using smartphones to access the Internet (Figure 6). Computer use was reported by 65% (n = 13) of interviewees, of these 70% (n = 9) worked at one of the two hospitals, the rest at a health centre. None of the dispensaries had computers, nor did the interviewees there have access to computers elsewhere. All participants stated that they were interested in computers and very interested in improving their computer skills. They all also thought ICT could be helpful in their work (see section on qualitative results).

### Qualitative data

This part addressed how the participants perceived the drug distribution chain in Tanzania today: if they had comments concerning the system and whether they thought ICT could be helpful in improving it. The emerging 5 themes are described below.

1. Medical Stores Department system.

2. Reported access to essential medicines and items in rural areas.

3. Perceived problems with the drug distribution chain at a district level.

4. Suggestions on how to improve today’s drug distribution chain.

5. Reported attitudes concerning ICTs role in CME.

### Medical stores department system

In 2002 Tanzania changed the drug distribution system to the so-called Integrated Logistics System (ILS), considered an improvement – albeit with limitations - by the informants. The main criticism of the system was the drug distribution monopoly of the Medical Stores Department (MSD) to public health facilities and the transfer of the yearly budget for drugs and medical equipment directly from the government to the MSD, leaving no alternative option for purchasing drugs or medical items when MSD was out of stock.

“We have improved, now the drug distribution chain is integrated and we can order different types of medicines in the same form” - # 2

“The distribution is somehow good, because we are now using ILS, but there are several areas with problems that needs improvement” – # 11

“The government does not plan or implement properly, that is why the system does not work. The government should be more active.” - # 3

### Reported access to essential medicines and items in rural areas

Stock-outs at MSD were highlighted by the participants as the major problem of the drug supply chain. Often half of the drug order would be missing upon delivery and certain essential drugs, e.g. metronidazole, were sometimes out of stock in subsequent orders. At some health facilities elective surgery had been cancelled because of missing items such as gloves and suturing equipment and at other facilities patients were told to buy and bring gloves in order to make the surgery possible. One facility reported to have been out of gloves for eight months. Another problem was drugs already expired or close to expiry date upon arrival. One health care worker wanted more anti-malarial treatment options due to suspected resistance to artemisinine-lumefantrine. Orders and deliveries of drugs were done quarterly, but because of seasonal fluctuations in disease burdens of e.g. malaria, essential medicine went out of stock faster than expected at the health facilities.

“One problem with the drug distribution chain in Tanzania today is missing drugs and items. Sometimes when we miss an item we request it again in the next order, but it is still missing, they write missing item, what does it mean? Does it mean that the item is not there or that the MSD do not want to deliver it to us although we need it? When it says missing item or out of stock at MSD, we have no alternatives.” - # 20

### Perceived problems with the drug distribution chain at a district level

Usually the clinician responsible for ordering drugs had one week to fill in the order form and thereafter had to personally bring it to the DMO office in Bunda or Serengeti, which caused difficulties. The person submitting the form was often the only employee at the health facility and as the trip could stretch over a couple of days there would be no one to care for the patients at the health facility in the meantime. Sometimes the responsible person was unable to order the drugs, e.g. because they did not know the procedures, because of illness, poor road conditions or because he/she could not afford the transportation to the DMO office. Delivery delays from the MSD to the district hospital were rare. However, the distribution from hospital to dispensary or health centre was often delayed, reportedly because of lack of a car, a driver, fuel or poor roads. Health facilities close to the hospitals rarely experienced delays, whereas dispensaries in remote villages reported delays ranging from a couple of weeks to months.

“Most of the clinicians responsible for ordering the drugs do not know the ordering procedure. They do not know how to fill out the forms correctly and they fail to bring them in time, this is a challenge.” - # 12

“It is difficult for me to leave the order, last week they called me and said they needed the order, but there was no money to bring the form from here to the hospital, we have to use our own money and we only have one vehicle in another village and it is often already gone when you get there.” - #18

“An easier way of ordering is needed” - # 8

### Suggestions on how to improve today’s drug distribution chain

To improve drug distribution, informants suggested that MSD should focus more on essential medicines, that systems to deal with several suppliers should be developed in order to avoid stock-outs, and that the government should become more active in supervising the MSD.

The current drug ordering system was perceived to require high levels of time and effort. Most informants could see immediate benefits of an electronic system with direct communication. They expressed a wish for more direct contact with the MSD to know available medicines in advance of placing an order and also to be able to give MSD feedback. Computers connected in a network were seen as the best ICT option for this. However, many health workers imagined mobile phones would be useful, as phones are well distributed and computers scarce. At district level computers and an electronic communication between the health facilities were desired in order to track drugs and get stock reports, and thus be able to redistribute drugs in time if needed. All interviewees but one (see quotation below) said that ICT would be helpful for purposes such as ordering of drugs, reduce the stock outs and delays.

“I do not think ICT could help as long as the government does not become more active.”- # 3

“ICT would help project the required amount of drugs you want and provide us with the baseline on which we would rely to make all of the drug orders. ICT would give opportunity to get stock reports on a daily basis, so if there was a stock-out in a health facility it would be easy to relocate and make sure that people get service. A computer network of some kind would be good because than many people would get access to similar information at the same time, have an easy self monitoring and get information of what is available at the MSD.”- # 1

“Computers would be better than mobile phones, but it requires electricity and skill.” - # 9

### Reported attitudes concerning ICTs role in CME

There was generally a positive attitude to computers and Internet for educational matters; everyone saw a possibility to improve their medical knowledge and skill. For many it would replace or add to yearly trips to Mwanza to attend seminars for updated medical information and education.

“Internet could give us new information about drugs and diseases without having to move from here to get that information.” - # 15

“To expand your knowledge; read and exchange ideas, instead of being isolated, to know what’s going on in the rest of the world.” - # 20

### Drug management application demonstration

No participant had seen or used an Android tablet before. Despite the lack of previous knowledge of this technology, the informants quickly understood the purpose and structure of the application and the touch-screen function. All interviewees were positive to the application although one participant found it difficult to accept that the tablet automatically calculated drug use and need in the same way that they were used to calculating it by hand. All interviewees were interested in training to use the tablet and they stated it could help improve the drug ordering system. Most informants also expressed a wish to be able to get access to drug stock information at MSD in real-time.

## Discussion

We report problems of drug shortage and challenges in drugs procurement in two rural districts in Tanzania. Mobile phones were widely used and the interest for ICT tools was great. Access to current medical information and CME was poor. Health workers shown a mobile application for drug ordering and tracking drug use were very positive about it and saw its potential in reducing drug stock-outs.

Severe shortage of medicines was reported as the main problem with drug distribution in this area. Even though the drug distribution system has few steps, leaks and severe delays were reported, but the leaks are difficult to trace and the magnitude of the problem difficult to estimate [19]. Difficult conditions, such as poor roads, lack of stable electricity and funding for fuel and transportation contributed to delays in drug orders and delivery. An estimated one fifth of patients did not get the 1^st^ or 2^nd^ line treatment needed and 8 of 20 health workers reported stock out of 1^st^ line treatment every week. The drugs and items missing in the deliveries from MSD were substantial and had not improved from March to December 2011 [19]. Similar to our findings, a study from 2007 reported that only 61-68% of medicines orders from hospitals and 67% of those from primary health care facilities were fulfilled [20]. The same study also reported that the actual spend on drugs in 2005/2006 in Tanzania was only 76% of the allocated budget, suggesting that improvement in drug procurement is needed even at central government level. As discussed by others health sector governance is an important factor in access to medicines [32,33].

There are several possible and acknowledged weaknesses and problems within the current Tanzanian distribution system that could explain the lack of access to essential drugs. The persons at the health facilities responsible for the ordering may not be fully aware of how to perform that task correctly and, as a consequence, do not manage to estimate how much of a certain drug is needed. Fluctuations in disease burden and the lack of any extensive disease surveillance make such predictions hard. The MSD drug distribution monopoly, like any other monopoly, does not have any competition and therefore no pressure to improve for survival. The consumer, in this case the public health facilities, has no other option other than relying on a weak distributor that does not deliver the requested supply [20]. There is anecdotal evidence of corruption and counterfeit drugs within the health care system in Tanzania, but the current drug distribution system does not include any possibility of tracking drugs to discover potential leaks [3,8,34]. Moving from paper-based to electronic systems can help improve transparency, and thereby possibly drug supply management [35]. Our informants claim that the Government does not sufficiently monitor or evaluate the MSD to see that they deliver the service they promise. Therefore simple tools to monitor drug use based on Defined Daily Doses and adherence to essential drug recommendations should be considered since this method makes it possible to easily compare drug use and drug distribution over time in an individual health-care unit and between units [36]. It has been shown that these simple methods can improve quality in drug use and prescribing in different healthcare settings [36]. Another factor that adversely affects the drug distribution in many low-income countries, is the number of disease specific (e.g. malaria, TB and HIV) vertical programmes that each deliver drugs for their intervention. This leads to a number of parallel systems and a very complex drug delivery chain system [35]. The aim of our app is to make it possible to integrate the supply chain for all diseases in one common delivery system, starting with those that are supplied through MSD.

Earlier reports urge African countries to identify leaks in the distribution chain and the Tanzanian government acknowledges that corruption and counterfeit drugs are a concern [3]. According to the MSD homepage they are cooperating with Danish International Development Agency (DANIDA) in developing an electronic system incorporating order to delivery and inventory management [18]. No representatives from MSD were interviewed in this study, however, MSD official documents state that they currently provide a full management information system for information about order statuses, inventories and deliveries [37]. Regardless of whether or not such a system existed, it did not effectively work in our rural study districts.

In our study healthcare staff asked for an electronic ordering procedure, as well as a redistribution system for all essential drugs similar to the recently implemented SMS for life for anti-malarial drugs [15]. Through this system facilities with small quantities of antimalarials in stock can receive more drugs straight from neighbouring facilities that have more drugs in stock [15]. A previously reported challenge regarding the supply chain management is this lack of communication between the final steps of the distribution system, and between health facilities located close by each other [35]. Our app currently communicates between all the different steps in the supply chain, and could also include communication between health facilities. Today, there are well-developed smartphones and tablets with health applications that can store a great deal of data and could therefore be suitable ICT tools for implementation. Although mobile coverage for voice calls and SMS exists almost everywhere, larger data volume capacity is mostly available in urban areas [23]. Simple prototype applications for ordering of essential medical supplies and reports about stocktaking have been developed and could be tested in pilot studies [30,38]. Similarly, a recent study has shown the potential of an android application in helping health workers optimise drug dosage schemes for antiretroviral therapy [39].

Our study participants were positive about ICT tools in CME. One telemedicine pilot study concerning HIV and antiretroviral therapy used e-mail/Internet discussion forums to provide CME for clinicians in resource-strained countries. The researchers successfully incorporated use of Internet and e-mail into the clinicians’ daily work. The forum was shown to be cost-efficient and effective and the users reported a great need for medical support and CME [13]. The availability of updated medical information in our study was scarce, especially in the most rural parts of the districts. Previous studies have shown that healthcare can improve if health workers have access to updated medical information and therefore lack of it may result in an inadequate care for the people, as the health workers do not follow the constantly changing medical treatment guidelines [40]. The Internet is a great source for medical research and information, if the user is taught how to use it properly and with a critical eye. These results are in line with findings from Uganda [41] and underline the potential of prompt access to drug information provided electronically and through medical books as reported by most interviews.

Every participant owned at least one mobile phone and as many as 8 out of 20 surfed the Internet through it. Although this was mainly in Bunda or Mugumu towns it was still a surprisingly high number considering the general low use of advanced technical devices. Access to 2G and 3G networks is possible because of big investments from mobile operators in resource poor countries during the last years [42]. According to The World Bank the number of mobile phone subscribers in Tanzania per 100 people has increased from 1 to 40 in the last ten years [9]. In our, as well as in other, studies [12,43], the mobile phone was an important communication tool for consultation and contact with patients, especially in the more rural parts where the mobile phone was the only communication tool available. Therefore the digital divide also results in a medical knowledge divide [40]. In our study 78% of the participants at the hospitals used Internet through their phones and 100% of the hospital health workers had some computer and Internet knowledge, still there was a distinct digital divide concerning access and skill in the two towns compared to the villages. A study from Ghana from 2000 [44] surveyed ICT knowledge among health care workers at one urban and one rural hospital. At the rural hospital most comparable to our setting, 78% of staff had knowledge of computing and 33% knew how to use the Internet. In this study, however, no participant at the rural hospital had a mobile phone with Internet function, showing a growing ICT development and interest among health care professionals in the past ten years in SSA [9].

Our respondents were generally very positive about the android tablet, and saw few limitations to its use. However, as with many ICT tools in this setting, providing access to support services and hardware repair is a challenge. New staff also need to be trained in the intervention and follow-up is needed. The positive attitude towards technology and the willingness to learn from end users is of paramount importance for future ICT projects in rich as well as in poor countries. To ensure ICT tools are user-friendly, sustainable and integrated into the daily routine of the health facility, the users need to be involved in the development process and implementation of technical tools [45]. It is important to define key factors that can help ascertain that ICT-tools are valuable in clinical practice [46]. In high-income countries it has been found that use of ICT-tools is highly linked to perceived usefulness and ease of use in daily clinical work [46,47]. Most likely the same factors are key components determining how ICT-tools are adapted in rural healthcare settings in Africa, but little work has been done to study this systematically. The findings from our pilot study have led to updates of the tablet application and work is no ongoing to test it with live data in Bunda and Serengeti districts.

One weakness in the study can be a potentially biased sample due to interviewing selected key informants and following with snowball sampling. However, this is a common and well-documented approach in qualitative research. Another potential weakness is the predetermined sample size of 20 respondents. Saturation may not have been reached during data collection, but we still think the data from our pilot study give a fair picture of the opinions of the health workers involved in the drug supply chain in the study districts. The android tablet application was presented in an English language version only. A Kiswahili version might have made it even easier to understand, and even more attractive to the participants. A translated version should be used for testing the application. In retrospect, with access to the information that emerged during the data collection, persons from the MSD should have been interviewed in the study to obtain a better overall perspective of the drug distribution system. We found very few other articles or information about ICT for securing drug distribution as well as ICT use and skill among health care professionals in resource poor countries, making our study an important contribution to the field.

## Conclusions

The potential for using ICT tools in resource-scarce settings is rapidly increasing as broadband and mobile technologies spread. Our pilot study shows great interest in and possibility to use ICT tools for improving ordering and stock keeping of drugs among health care workers in a rural, poor area of Tanzania. An electronic drug ordering system would save time and effort, reduce transportation costs for the users and minimise drug stock-outs. Furthermore, the android tablet could be used to reach out with CME programs for health care workers at remote health facilities, as well as those in towns. This could boost the medical knowledge of employees on all levels of the health system and hopefully improve patient care. A further test of the drug ordering system and evaluation of its impact on health facility drug availability in collaboration with MSD is needed.

## Competing interests

The authors declare that they have no competing interest.

## Authors’ contributions

JE, BP, LG and AN conceived of the study, and participated in its design. All authors participated in the design of the study. JN conducted the data collection. JE coordinated the data collection whereas DM and AN assisted in coordinating the field work. JN, PBR, JE and LG performed the data analysis. JN and JE drafted the manuscript and JE coordinated its finalization. All authors read and approved the final manuscript.

## Pre-publication history

The pre-publication history for this paper can be accessed here:

http://www.biomedcentral.com/1472-6947/14/78/prepub

## Supplementary Material

Additional file 1 Interview guides. Click here for file
