# Supplementary material for: A cross-sectional pilot study assessing needs and attitudes to implementation of Information and Communication Technology for rational use of medicines among healthcare staff in rural Tanzania
Source: BMC Med Inform Decis Mak. 2014 Aug 27;14:78. doi: 10.1186/1472-6947-14-78 (PMC4164118; doi:10.1186/1472-6947-14-78)
Supplement: Additional file 1 — Interview guides. [file 1472-6947-14-78-S1.pdf]

Additional file 1 – interview guides

Semi structured interviews

(All interviewees)

Nr:

Date/time:

Interviewer:

Interpreter:

Interviewees:

1) This interview was conducted in

a. Location: ________________________________

b. Language: ______________________________

2) Which language(s) do you use at work?

3) Age: ___________

4) Gender Male Female

5) Highest education achieved (if applicable): ____________________________________

6) Specialization (if health care worker): ____

7) Current position

a. _______________________________

b. Type of health facility (if applicable): ______________________________

c. Name of health facility (if applicable): ______________________________

d. Name and type of other workplace (if applicable): ____________________

8) How long have you had the present position?

a. < 1 year

b. 1-5 years

c. > 5 years

9) What Information and Communication technology (ICT) do you use privately or/and in your work?

a. Telephone Privately / At work

b. Mobile Phone Privately / At work

c. Computer Privately / At work

d. Internet Privately / At work

e. TV	Privately / At work
f. E-mail	Privately / At work
g. Fax	Privately / At work
h. Other (specify):	Privately / At work

10) For what purpose do you use a telephone / mobile phone?

a. Private use

b. Teleconferences

c. Consultation

d. Communication with patients

e. Disease surveillance

f. Education

g. Connect to internet

h. E-mail communication

i. Other (specify):

11) If you use computers: where do you have access to a computer?

a. At home

b. At work

c. Through an acquaintance

d. At a store/cafe

e. Other

f. I don't have access to a computer

12) If you use computers: what kind of computer(s) do you use?

a. Desk based

b. Laptop

c. Both

13) If you use computers: for what purpose do you use a computer?

a. Social media

b. Search engines

c. Internet

d. Education

e. E-mail communication

f. Administrative work

g. Patient recording

14) If you use computers: how often do you use a computer?

a. Daily

b. Several times a week

c. Once a week

d. Several times a month

e. Once a month

f. Less than once a month

g. Never

15) Are you interested in computers?

a. Yes

b. No

16) Do you think ICT (examples in question 9) could help you in your work?

a. Yes

b. No

c. Don't know

17) How interested are you in improving your computer skills?

a. Very interested

b. Interested

c. I don't know

d. Not so interested

e. No t interested

18) How would you describe yourself as a computer user?

a. No experience

b. Very inexperienced

c. Inexperienced

d. Some experience

e. Experienced

f. Very experienced

To Head of health facility

19) Do you have electricity at your health facility?

a. Yes

b. No (If no, skip to question 23)

20) How often do you experience power cuts at work?

a. Daily

b. Several times a week

c. Once a week

d. Several times a month

e. Once a month

f. Less than once a month

g. Never

21) How long does the power cuts usually last?

a. Less than an hour

b. A couple of hours

c. For a day

d. Several days

e. For a week

f. Several weeks

g. For months

22) Do you have access to stand-by generators, solar energy or any other source of electricity at the facility/workplace?

a. Yes

b. No

c. Don’t know

23) How many computers are there in the health facility or in the office were you work?

Specify number: ____________

a. How many of them are installed and ready to use?

b. Who’s got access to them?

i. Chief of clinic

ii. Administration

iii. Clinicians

iv. Other (specify):

c. Do you have available technical support for the computers?

i. Yes

ii. No

iii. Don’t know

d. What are the computer(s) used for today?

i. Personal use (e.g surfing the Internet)

ii. Education

iii. Administration

iv. E-mail, communication

v. Patient records

vi. Distance consultations

vii. Other (specify):

24) Do you have the financial possibility to buy computers to your health facility today?

a. Yes

i. If yes, how many?

b. No

c. Don't know

To CO, AMO, MO

25) How do you get continuous medical education?

(Meaning further education after you finished education and started working)

a. I don't get any continuous medical education

b. Meetings

c. Medical journals

i. If yes: where do you get these?

d. Medical literature/books

i. If yes: where do you get these?

e. Medical newsletters

i. If yes: where do you get these?

f. Internet

g. Workshops/ Conferences

h. Courses

i. TV - videos

j. How often do you use any of these sources?

26) Do you receive new guidelines regarding disease management?

a. Yes

i. If yes, from where?

b. No

27) What interest do you have in getting continuing education through video conferences?

a. Very interested

b. Interested

c. Maybe interested

d. Not interested

28) What interest do you have in being able to consult specialists/other clinicians through video conferences?

a. Very interested

b. Interested

c. Maybe interested

d. Not interested

29) Out of ten patients, for how many do you have access to the 1st or 2nd line treatment necessary to treat them?

Please specify number: ________________

30) How often do you experience that you are out of stock of at least one 1st line treatment at your health facility?

a. Every day

b. Several times a week

c. Once a week

d. Several times a month

e. Once a month

f. Almost never

g. Never

31) How often do you have access to essential (1st or 2nd line treatment) medicine for:

a. Malaria

Always / Mostly / Rarely / Never / Don’t know / Don’t treat

b. TB

Always / Mostly / Rarely / Never / Don’t know / Don’t treat

c. HIV/AIDS

Always / Mostly / Rarely / Never / Don’t know / Don’t treat

d. Pneumonia

Always / Mostly / Rarely / Never / Don’t know / Don’t treat

e. Diarrhea

Always / Mostly / Rarely / Never / Don’t know / Don’t treat

f. Pain/fever (Analgesics)

Always / Mostly / Rarely / Never / Don’t know / Don’t treat

Qualitative Questions

First section with probes for the following interviewees:

District medical officer (at the hospital, responsible for ordering drugs)

District Pharmacists (at the hospital)

District executive director (district headquarters, other relevant person?)

1) What do you think of the drug distribution chain in Tanzania today, do you see any problems? If so, do you have any suggestions for improvement?

2) How do you think that ICT applications could be helpful?

For example in:

a) Electronic ordering of drugs?

i. How do you think the ordering of drugs could be developed?

E.g. - Using email orders

- Using computers to a network

- Using mobile phones

b) Continuous medical education?

(If yes, how? If not, why not?

Do you have any suggestions on how they could be implemented?)

3) Are there any ongoing ICT projects for public health in your district?

This section with probes for clinical officers, assistant medical officers and medical officers:

1) What do you think of the drug distribution chain in Tanzania today, do you see any problems? If so, do you have any suggestions for improvement?

a) How reliable is the delivery of the drugs to your health facility?

E.g. - Do you always get drugs on time?

- Do you always get the drugs you order?

2) How do you think that ICT applications could be helpful?

For example in:

a) Electronic ordering of drugs?

i. How do you think the ordering of drugs could be developed?

E.g. - Using email orders

- Using computers to a network

- Using mobile phones

b) Continuous medical education?

(If yes, how? If not, why not?

Do you have any suggestions on how they could be implemented?)
